# A Multicompartment Approach - Diatoms, Macrophytes, Benthic Macroinvertebrates and Fish - To Assess the Impact of Toxic Industrial Releases on a Small French River

**DOI:** 10.1371/journal.pone.0102358

**Published:** 2014-07-14

**Authors:** Manon Lainé, Soizic Morin, Juliette Tison-Rosebery

**Affiliations:** UR EABX, Irstea, Cestas cedex, France; Catalan Institute for Water Research (ICRA), Spain

## Abstract

The River Luzou flows through a sandy substrate in the South West of France. According to the results of two assessment surveys, the Water Agency appraised that this river may not achieve the good ecological status by 2015 as required by the Water Framework Directive (2000/60/EC). This ecosystem is impacted by industrial effluents (organic matter, metals and aromatic compounds). In order to assess and characterize the impact, this study aimed to combine a set of taxonomic and non-taxonomic metrics for diatoms, macrophytes, macroinvertebrates and fish along the up- to downstream gradient of the river. Diversity metrics, biological indices, biological and ecological traits were determined for the four biological quality elements (BQE). Various quantitative metrics (biomass estimates) were also calculated for diatom communities. The results were compared to physicochemical analysis. Biological measurements were more informative than physicochemical analysis, in the context of the study. Biological responses indicated both the contamination of water and its intensity. Diversity metrics and biological indices strongly decreased with pollution for all BQE but diatoms. Convergent trait selection with pollution was observed among BQE: reproduction, colonization strategies, or trophic regime were clearly modified at impaired sites. Taxon size and relation to the substrate diverged among biological compartments. Multiple anthropogenic pollution calls for alternate assessment methods of rivers' health. Our study exemplifies the fact that, in the case of complex contaminations, biological indicators can be more informative for environmental risk, than a wide screening of contaminants by chemical analysis alone. The combination of diverse biological compartments provided a refined diagnostic about the nature (general mode of action) and intensity of the contamination.

## Introduction

Rivers are a key component for the development of civilizations and, therefore, human-induced impacts on these ecosystems are diverse and always increasing. Since 2000 in Europe, the Water Framework Directive [1] requires water bodies to achieve good ecological status by 2015, and consider biology as the central element of the assessment. This Directive recommends using four biological quality elements (BQEs) – diatoms, macrophytes, benthic macroinvertebrates and fish – to assess the rivers' ecological status.

The River Luzou flows through a sandy substrate in the South West of France (Landes). From the results of two assessment surveys (2004 and 2006), the Water Agency appraised that this river may not achieve the good ecological status by 2015 [2]. The ecosystem is impacted by the effluent of an industrial plant producing rubber: the pollution is very diverse (mainly organic matter, metals and aromatic compounds including aniline), variable in composition over the year, and released in pulses (51 releases in 2010).

In this context of abundant, complex, toxic pollution, the question is how to characterize the ecological status of the river Luzou? In France, river assessment is based on several biological indices: IPR (Indice Poisson Rivière) for fish fauna [3]–[4], IBMR (Indice Biologique Macrophytes Rivières) for macrophytes [5], IBGN (Indice Biologique Global Normalisé) for benthic macroinvertebrates [6] and IBD (Indice Biologique Diatomées) for diatoms [7]–[8]. While these indices are mostly sensitive to trophic pollution, recent works in Europe focused on methods to assess multi-stress conditions: IPR+ [9], I2M2 [10], SPEARorg [11] or SPEARpest for phytosanitary disturbance [12]. Usually, the four BQEs are studied separately but it has already been demonstrated that to assess ecological status of rivers, they are complementary [13]–[16].

Nevertheless, even when combined, indices often lead to excessive reduction of environmental information [17], so the use of non-taxonomic measures such as biological and ecological traits provides new perspectives for biological assessment methods [18]–[19]. Traits represent qualitative and quantitative information related to the biology of organisms and their relationship with the environment. Traits describe taxon ecological preferenda, life cycle, morphology, physiology or behaviour [12], [20]–[25].

In this context, we hypothesized that the assessment of the ecological status of the River Luzou according to the recommendations of the WFD, e.g. biological indices calculation and punctual physicochemical analysis of the water, would be insufficient to highlight the impact suffered by the studied ecosystem. In other words, we assumed that the combination of relevant taxonomic and non-taxonomic metrics for the four BQEs cited along the up- to downstream gradient of the River Luzou, is a consistent way to assess the impacts of the industrial releases on the ecosystem. We tested our hypothesis by sampling benthic macroinvertebrates, diatoms, macrophytes, fish and water at three sites on an up- to downstream gradient of the river, in different seasons, in 2009 and 2010. We compared the relevance of the different types of results, e.g. classical biological indices, taxonomic and non-taxonomic metrics, punctual physicochemical parameter values, to accurately monitor the changes in major ecosystem components.

## Materials and Methods

### Study area and sampling sites

**Figure 1:**
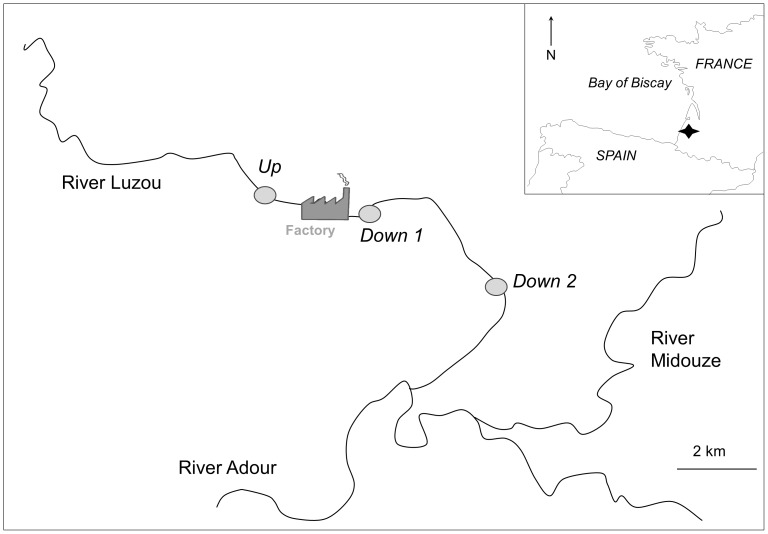
Study area and sampling sites.

The River Luzou (Landes, South West France) is a small river (28 km long) flowing through a sandy substrate, and characterized by low pH, low conductivity and low nutrient concentrations (Fig. 1). The industrial plant, situated about 18 km from the river source, introduces highly toxic pollution and in addition increases the river water temperature through use of water for cooling and tank washing processes. The wastewater is stored in a settling pool and overflow is released into the River Luzou directly downstream of the plant. Three sampling sites publicly accessible were selected along the study reach: a site called “Up” situated upstream of the factory (Lambert 93 coordinates: X385686, Y6312556) was considered as the reference station (i.e. unimpacted by the toxic effluents); sites “Down 1” (X387414, Y6312572) and “Down 2” (X389923, Y6310416) were situated about 500 m and 4500 m downstream of the factory, respectively. Benthic macroinvertebrates, diatoms, macrophytes, fish and water were sampled at the three sites once or several times, in different seasons, in 2009 and 2010 (see below for details). The water, macrophyte, macroinvertebrate, and phytobenthos sampling procedures were approved by the Adour-Garonne Water Agency.

### Physicochemical parameters

Temperature, pH, conductivity and oxygen saturation were measured *in situ* with appropriate WTW probes. During diatom samplings at day 30, two litres of water were collected from the main flow area near the middle of the river and kept at 4°C for analysis within 24 h, according to AFNOR standardized protocols. The following parameters were analyzed by an accredited laboratory: total suspended particulate matter (SPM), biological oxygen demand (BOD), and concentrations of sulfates (SO_4_^2−^), ammonium (NH_4_^+^), nitrites (NO_2_^−^), nitrates (NO_3_^−^), Kjeldahl nitrogen (Nkjeldahl), phosphates (PO_4_^3−^), total phosphorus (PT), carbonates (HCO_3_^−^), calcium (Ca), chlorine (Cl), heavy metals (copper, lead, aluminium, iron, manganese, nickel, zinc, cadmium, mercury), cyanide, arsenic, pesticides (142 substances, including degradation products), benzene and nitrobenzene, toluene and 2-nitrotoluene.

The substances monitored were chosen because they are known to be present in the releases, as stated in the self-monitoring data from the plant itself. Parameters showing values below the detection limit for all upstream and downstream sites were removed from the analysis. For those occasionally below, half the value of the detection limit was used [26].

Additionally, maximal theoretical concentration of aniline (a target component of the toxic wastes released by the industrial site) was evaluated using industrial monitoring data from year 2010 and the QMNA5 value (monthly low flow value that may not occur more than once every 5 years).

### Fish sampling

Ethics statement: All fish were properly collected and handled in an ethical manner, with all required permissions from the Adour-Garonne Water Agency. No other permissions were required for completion of this research, and this study does not include endangered or otherwise protected species.

Fish surveys were carried out by experienced fisheries staff of the departmental fishing federation AAPPMA64 (accredited by ECCEL Environnement) and all sampling procedures complied with the French and European Union legislation on animal welfare. Electric fishing was carried out at the minimum power settings needed to incapacitate the fish and thus no adverse impact was expected. The fish were handled with great care. This includes electrofishing and manipulations (counting, weighing and measurements) where fish were maintained in river water. After measurements, all fish were returned alive into the river.

All sites were electrofished (pulsed direct current waveform), during low flow period (September 2009) and according to the NF EN 14011 standard [27]. Electrofishing was authorized by the Adour Garonne Water Agency and performed by operators accredited by ECCEL Environnement. Fish were sorted and stored in a large basin, and then counted, measured and weighed, then released alive in the water. In the case of very numerous individuals, fish were counted and weighed in homogenous sets. The sampling area was systematically reported.

### Macrophyte sampling

All sampling was carried out according the standard NF T90–395 [5] during vegetation periods (September 2009, July 2010). The areas covered by macrophyte beds and by each taxon were evaluated for each site. Taxa difficult to identify *in situ* were collected, packed and transported to the laboratory for determination.

### Benthic macroinvertebrate sampling

Macroinvertebrates were collected at each of the 4 seasons (Autumn: September 2009, Winter: November 2009, Spring: April 2010 and Summer: July 2010). Macroinvertebrates were collected with a Surber sampler (mesh size 500 µm, sampling area 0.05 m^2^), the device required by the French standardized protocol XP T90–333 [28]. Micro-samples were taken at each site from among twelve mesohabitats defined as visually distinct units within the stream, considering apparent physical uniformity (*sensu* Armitage et al. 1995), and described by a combination of substrate types and current velocities. Mesohabitats were sampled in a hierarchical order according to the IBGN standard [6] to maximize the taxonomic richness of the faunal assemblage at the site scale, after gathering sample units. Macroinvertebrates were sorted and identified to the family level following standard XP T90–388 [29], except for some groups identified at a higher taxonomic level (i.e. *Oligochaeta*, *Bryozoa*, *Nematoda*, *Hydracarina*). The twelve micro-samples were pooled to finally constitute a single sample, used to calculate the IBGN index [6].

### Phytobenthos sampling

Phytobenthos was also sampled four times (Autumn: September 2009, Winter: November 2009, Spring: April 2010 and Summer: August 2010).

#### Quantitative measurements

Artificial substrates were immersed in the river to quantify dry weight, ash-free dry mass of biofilms, chlorophyll *a* concentrations, and the number of live and dead diatom cells. Six glass slides (total surface area reaching 300 cm^2^) were placed in a rack at the three sites [30]. After 15 days immersion (t15) and 30 days immersion (t30), three slides were removed from the water and scraped into a standard volume of mineral water, to obtain three replicates per sampling date then separated into aliquots.

A 20-mL aliquot was used to determine the dry weight (DW) and ash-free dry mass (AFDM) of the biofilm, expressed as mg cm^−2^, according to European standard NF EN 872 [31]. Ten millilitres of the suspension were filtered through a Whatman GF/C filter, then extracted with acetone for 24 h before spectrophotometric analyses. Chlorophyll *a* concentrations were calculated following Lorenzen [32]. A 5-mL aliquot was preserved with 0.5 mL of formalin solution for diatom cell density enumeration [live and dead, 33] and taxonomic identification.

#### Diatom community characterization

Diatom samples were collected from natural surfaces (pebbles or macrophytes, at t30) and artificial substrates (at t15 and t30), according to a standardized method NF T 90–354 [7]. For each slide, 400 valves were determined to the lowest taxonomical level possible. Diatom species were identified at 1000× magnification (Leitz DMRB light microscope), mainly according to Krammer and Lange-Bertalot [34] and Lange-Bertalot [35], by examining permanent slides of cleaned diatom frustules, digested in boiling H_2_O_2_ (30%) and HCl (35%) and mounted in a high refractive index medium (Naphrax, Northern Biological Supplies Ltd., UK; RI  = 1.74). A total of 234 diatom taxa were identified. The 107 taxa with abundances higher than five individuals (considering all samples) were used to describe community structure.

### Taxonomic and indicial metrics

For the four biological compartments and for all sampling dates, specific richness (S), Shannon diversity (H) [36] and Pielou equitability (J) [37] indices were calculated, as well as the French biological indices used for biomonitoring: IBGN [6], IBD [7], IPR [3], [38] and IBMR [5]. In addition, for benthic macroinvertebrates SPEARpesticides [12] and SPEARorganic [11] indices were determined thanks to an online application (http://www.systemecology.eu/spear/). Finally, for diatoms the polluosensitivity index IPS [39] and the occurrence of teratogenic forms were calculated based on the 234 taxa.

### Biofilm-related quantitative metrics

Seasonal variations in biomass: dry weight, ash-free dry mass, chlorophyll *a* concentrations, and the number of live and dead diatoms were reduced by normalization using the mean values calculated on the reference site (Up) at each season.

### Functional metrics

For each taxon within each biological compartment, different functional traits were listed from the literature. Each trait shows at least two modalities, and for each modality a score is assigned to the taxon. A Taxa × Traits table was obtained for each compartment. Fish traits are listed from Keith and Allardi [20]: trophic guilds, nesting substrates and position in the water column. Affinity scores were based on a 0 (“no affinity”) to 1 (“affinity”) scale. Considering benthic macroinvertebrates, eleven biological traits and eleven ecological traits were determined from Tachet et al. [40]. Affinity scores were scaled from 0 (“no affinity”) to 5 (“high affinity”). Diatom traits were derived from the Irstea database (https://hydrobio-dce.cemagref.fr/) for biovolumes and pioneer forms, Kelly et al. [41] for growth forms and Passy [22] for guilds. Affinity scores were scaled from 0 (“no affinity”) to 1 (“affinity”). For macrophytes, floristic groups -phanerogams, algae, bryophytes and heterotrophs- (scores from 0 “no affinity” to 1 “affinity”) were assigned to taxa and biological phanerogam traits (scores from 0 “no affinity” to 2 “strong affinity”) were listed from Willby et al. [25].

### Data analysis

Major differences between sampling sites considering physicochemical parameters were investigated using Principal Component Analysis (PCA). Data were normalized and redundant variables identified through Spearman pairwise correlation tests. Among redundant variables, only one was kept. Concentrations of aniline were taken as supplementary variable, assuming that the upstream site (Up) was free of aniline.

Concerning biological data, diatom and macroinvertebrate community structures were described using PCA based on species relative abundances. Functional traits-related information was obtained from the tables Taxa × Samples and Taxa × Traits for each compartment. Taxa × Samples tables were expressed in abundances, except for macrophytes (percentage cover) and fish (biomass). In order to produce functional profiles for each trait (relative distribution of the information among the categories), the following process was applied: i) for a given site and for each category of traits, taxon scores were weighted by abundances (cover or biomass); ii) the sums of the weighted scores were then expressed as a relative abundance distribution (within a trait), giving the site trait profile. For diatoms, this analysis was based on the 107 dominant taxa.

Only diatoms and benthic macroinvertebrates enabled statistical analyses (four sampling dates). Taxonomic, indicial and functional metrics were compared between sites by Kruskal-Wallis tests. For macrophytes and fish (respectively two and one replicates), only simple visual comparisons were possible.

All analyses were performed using the R software, version 3.0.2 [42], with packages ade4 [43] for descriptive analyses of data and agricolae [44] for Kruskal-Wallis tests.

## Results

### Analysis of environmental data

Alkalinity and HCO_3_^−^ giving redundant ecological information (Spearman test  = 1), HCO_3_^−^ was removed from the dataset. The following parameters, even though they were known to be released in the effluents, were systematically below the detection limit and were thus removed from the analysis: copper, lead, manganese, nickel, cadmium, mercury, cyanide, arsenic, benzene and nitrobenzene, toluene and 2-nitrotoluene. For the same reason, pesticides were also removed.

The environmental parameters finally kept for analysis and their contributions to axes 1 and 2 are listed in Table 1.

**Table 1:** Average (±standard errors, n = 4) values and contributions of environmental parameters to axis 1 and axis 2 of the PCA performed on physicochemical parameters (see Figure 2).

Parameter	Code	Tox	Up	Down1	Down2	Axis 1	Axis 2
Aluminium (µg L^−1^)	Al	100	306±163	322±213	266±141	−0.38	*0.51*
Aniline (µg L^−1^)	Ani	2.2	b.d.l.	0.44	0.44	0.48	*0.19*
Calcium (mg L^−1^)	Ca		5.5±0.3	7.5±0.5	8.4±0.9	0.48	0.19
Chloride (mg L^−1^)	Cl		19.7±0.5	20.4±0.3	20.1±0.4	−0.01	*0.65*
Conductivity (µs cm^−1^)	Cond.		123±4	175±26	176±22	*0.88*	0.06
Biological Oxygen Demand (mgO_2_ L^−1^)	DBO5		1.3±0.2	4.9±2.2	2.9±0.5	*0.60*	0.00
Total iron (µg L^−1^)	Fe.brut	300	287±62	340±64	326±58	0.00	*0.56*
Total manganese (µg L^−1^)	Mn.brut		16±3	17±3	24±3	0.00	0.40
Total Suspended Matter (mg L^−1^)	SPM		11.0±9.6	3.9±1.7	4.0±1.6	−0.22	0.20
Kjeldahl nitrogen (mg L^−1^)	Nk		0.8±0.3	3.3±1.4	3.0±1.2	*0.82*	0.01
Ammonium (mg L^−1^)	NH4		0.1±0.0	2.3±1.2	2.3±1.2	*0.85*	0.01
Nitrates (mg L^−1^)	NO3		5.1±1.1	4.6±1.2	5.2±1.0	−0.47	0.46
Oxygen saturation (%)	O2sat		97.9±6.3	92.3±8.7	89.0±8.2	−0.46	0.08
pH	pH		6.8±0.3	6.5±0.3	6.1±0.4	0.09	*−0.8*
Total phosphorus (mg L^−1^)	Ptot		0.03±0.00	0.03±0.01	0.02±0.01	−0.41	0.03
Sulfates (mg/ L^−1^)	SO4		10.9±0.9	28.4±9.1	26.1±7.3	*0.88*	0.06
Temperature (°C)	Temp		12.4±0.9	12.7±1.4	12.7±1.3	*0.51*	−0.01
Alkalinity (°f)	TAC		0.9±0.1	1.4±0.3	1.5±0.4	*0.84*	−0.02
Total zinc (µg L^−1^)	Zn.brut	30	5.8±0.6	21.0±4.7	17.3±0.6	*0.67*	0.14
Dissolved zinc (µg L^−1^)	Zn.dis		4.8±1.2	13.5±2.4	13.8±1.4	0.18	0.39

Aniline data calculated *a posteriori* are provided for information, b.d.l. below detection limit. Toxicity benchmarks (Tox) are freshwater values from the Canadian Water Quality Guidelines for the Protection of Aquatic Life (http://www.pesticideinfo.org/). Italics indicate the variables significantly discriminant (cos^2^>0.50).

**Figure 2:**
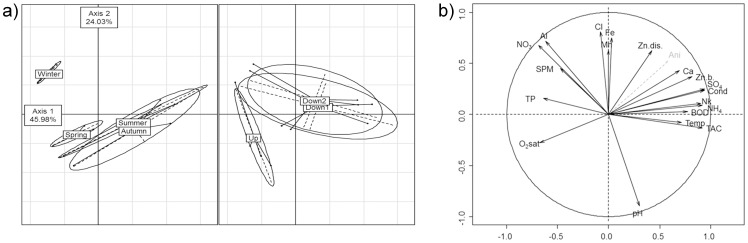
Principal Component Analysis based on physicochemistry. a) Principal Component Analysis performed on physicochemical data, with samples grouped by season (left panel) or by sampling site (right panel); b) Correlation circle. See Table 1 for abbreviations and contributions of environmental parameters on axis 1 and 2.

Axes 1 and 2 account for 70% of the total inertia (Fig. 2a). Axis 1 discriminates upstream from downstream conditions but does not clearly discriminate Down 1 from Down 2, while both Axes 1 and 2 separates winter samples from the others.

Downstream sites are characterized by higher concentrations of Zn, Nkjeldahl, SO_4_^2−^, NH_4_^+^, BOD5, higher conductivity, alkalinity and temperature (Fig. 2b).

Mean calculated concentration of aniline downstream was 0.44 µg L^−1^. Aniline, represented *a posteriori*, shows a cos^2^ equal to 0.42 on axis 1 and to 0.28 on axis 2.

### Analysis of biological data

Upstream communities were typical of the Landes ecoregion. Minnow (*Phoxinus phoxinus*), gudgeon (*Gobio gobio*), stone loach (*Barbatula barbatula*), brook lamprey (*Lampetra planeri*) and eel (*Anguilla anguilla*) were present in the River Luzou. The benthic macroinvertebrate fauna (thirty taxa) is dominated by *Chironomidae* and *Gammaridae*. Acidophilous and neutrophilous diatom taxa from the genera *Eunotia* and *Brachysira* were abundant, associated with numerous *Karayevia oblongella*, *Tabellaria flocculosa* and *Peronia fibula*. The macrophytic community was mainly composed of phanerogams characteristic of oligotrophic and acidic waters: *Myriophyllum alterniflorum* and *Potamogeton polygonifolium*.

### Taxonomical metrics and indices

**Figure 3:**
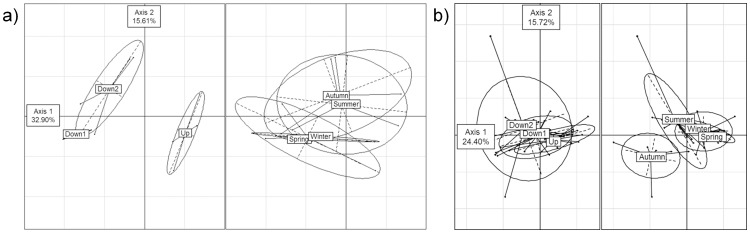
Contrasted responses of a) benthic macroinvertebrates and b) diatoms to seasonal and longitudinal changes.

Table 2 gathers information about differences between upstream and downstream conditions, according to Kruskal-Wallis tests. Taxonomic metrics based on fish, macrophytes and benthic macroinvertebrates showed similar responses to toxic pollution: a strong decrease in species richness, diversity and equitability was observed at station Down 1. Fish response was very marked, as only two minnow specimens were found at station Down 1 (considered as a null biomass), and a still low biomass was harvested at station Down 2. Macrophyte community structure was strongly shifted from a diversity of algae, bryophytes (e.g. *Fontinalis antipyretica*) and spermaphytes (in particular, *Callitriche platycarpa*), to a *Sphaerotilus* sp.-dominated community at Down 1, causing the dramatic decrease in taxonomic metrics observed. At Down 2, the diversity of taxonomic groups increased, and high percentages of algae (such as *Cladophora* sp.) were found. Taxonomic metrics for benthic macroinvertebrates were also significantly lower at stations Down 1 and Down 2, reflecting that macroinvertebrate species composition was mainly influenced by the up- to downstream gradient (Fig. 3a), more than by season (cold vs. warm waters). No significant differences between downstream and upstream sites were globally observed for diatoms, but the flora showed large seasonal variations (Fig. 3b).

**Table 2:** Taxonomic and quantitative metrics: calculation (mean and standard deviation) and differences between sites.

		Up	Down1	Down2	*P* value
**Fish**	S	6.00	*1.00*	8.00 *	-
	H	1.27	*0.00*	1.31 *	-
	J	0.70	*NA*	0.63	-
	Biomass	57.50	*0.00*	12.00 *	-
	IPR	14.62	*52.28*	22.49 *	-
**Macrophytes**	S	10.50±2.12	*4.00*±*1.41*	15.00±1.41	-
	H	1.22±0.07	*0.03*±*0.04*	2.02±0.03	-
	J	0.52±0.02	*0.02*±*0.02*	0.75±0.04	-
	IBMR	13.29±0.55	*3.22*±*3.49*	*10.93*±0.07 *	-
**Macroinvertebrates**	S	30.00±5.77	*10.00*±*3.37*	*18.50*±3.42 *	0.008
	H	1.95±0.43	*0.56*±*0.17*	*1.03*±*0.45*	0.02
	J	0.57±0.10	*0.24*±*0.05*	*0.35*±*0.15*	0.03
	IBGN	13.00±2.16	*3.50*±*1.29*	*8.50*±*0.58*	0.007
	SPEARp	35.05±2.77	27.37±5.66	34.95±4.55	0.08
	SPEARo	-0.54±0.04	*-0.68*±*0.02*	*-0.62*±*0.08*	0.04
**Diatoms - pebbles**	S	40.25±4.92	44.00±8.87	33.75±13.99	0.5
	H	2.73±0.42	2.88±0.50	2.36±0.86	0.61
	J	0.74±0.09	0.76±0.09	0.67±0.17	0.73
	TER%	0.24±0.34	1.65±1.20	1.00±1.41	0.18
	IPS	17.4±1.47	16.85±1.36	17.45±1.07	0.87
	IBD	19.95±0.10	18.68±1.65	19.38±0.43	0.3
**Diatoms - t30**	S	36.00±7.39	*46.25*±*8.02*	*49.5*±*6.61*	0.03
	H	2.72±0.46	3.06±0.20	2.98±0.28	0.58
	J	0.76±0.09	0.80±0.03	0.77±0.07	0.87
	TER%	0.13±0.25	0.37±0.14	0.19±0.24	0.36
	IPS	17.85±2.02	17.88±0.85	15.73±3.60	0.49
	IBD	19.93±0.15	20.00±0.00	18.95±2.10	0.57
	Live	1.00±0.18	*0.55*±*0.37*	*0.33*±0.25 *	0.0001
	Live/Dead	1.00±0.12	1.12±0.74	0.97±0.48	0.81
	DW	1.00±0.26	2.07±2.44	0.55±0.53	0.06
	AFDM	0.98±0.31	1.36±0.97	0.63±0.48	0.07
	Chlo.a	0.98±0.69	*0.38*±*0.58*	*0.31*±*0.28*	0.002
**Diatoms - t15**	S	42.00±7.53	48.00±7.87	45.25±11.00	0.72
	H	2.90±0.27	3.06±0.17	2.38±1.01	0.69
	J	0.78±0.07	0.79±0.05	0.62±0.23	0.38
	TER%	0.00±0.00	0.06±0.13	0.37±0.60	0.3
	IPS	18.50±0.90	17.80±1.05	15.23±2.55	0.07
	IBD	19.73±0.55	19.70±0.60	18.18±2.25	0.23
	Live	1.00±0.22	0.95±0.50	0.85±0.41	0.45
	Live/Dead	1.00±0.23	*0.69*±*0.48*	0.78±0.38	0.048
	DW	0.98±0.16	*2.88*±*1.87*	1.50±0.97	0.03
	AFDM	1.07±0.28	*3.43*±*3.23*	1.54±0.90	0.03
	Chlo.a	0.89±0.40	1.06±1.13	0.56±0.57	0.23

Italics indicate that the metric is significantly different from the reference station (Up), stars indicate significant differences between Down 1 and Down 2. Abbreviations: Specific richness (S), Shannon diversity (H), Pielou equitability (J), percentage of diatoms abnormal forms (TER%), dry weight (DW), ash-free dry mass (AFDM), chlorophyll *a* (Chlo.a), and biological indices based on macroinvertebrates (IBGN), fish (IPR), and diatoms (IBD and IPS). NA: Metric calculation not possible. Quantitative metrics for diatoms from artificial substrates (t15, t30) are normalized by Up values at each sampling date.

Poor IPR, IBMR and IBGN scores classified station Down 1 in a “bad” ecological status, and station Down 2 in a “medium” to “poor” status (Table 2). The high abundance (up to 60%) at downstream sites of *Achnanthidium minutissimum*, a species considered as oligo- to mesotrophic by IBD and IPS, maintained good index scores whereas the SPEARorganic index differentiated upstream and downstream sites.

Diatom communities sampled on glass slides 30 days after immersion presented a strong decrease in live cell density and in chlorophyll *a* concentrations downstream. At station Down 1, dry weight and ash-free dry mass increased for communities sampled on glass slides 15 days after immersion, but diatom mortality was significantly higher (decrease in the ratio of live to dead cells).

### Functional metrics

**Diatoms.** Even though Kruskall Wallis test results were not significant, some functional traits from communities sampled on natural substrates (pebbles) showed trends between upstream and downstream sites (Table 3). Pioneer species were more abundant at station Down 2 while “high profile” and “motile” species decreased. Downstream, high biovolume taxa regressed in favour of smaller ones (<99 µm^3^). No particular upstream-downstream pattern emerged from growth forms.

**Table 3:** Diatom-related traits: calculation (mean and standard deviation) and differences between sites.

	Trait	Modality	Up	Down1	Down2	*P* value
**Diatoms - pebbles**	Pioneer forms	Non-pioneer	95.78±4.31	75.66±13.27	56.67±29.22	0.09
		Pioneer	4.22±4.31	24.34±13.27	43.33±29.22	0.09
	Growth forms	Adnate	2.97±1.29	1.24±0.49	1.59±1.52	0.15
		Pedunculate	19.58±9.08	28.16±4.71	36.46±11.77	0.09
		Colonial	38.65±17.73	28.75±15.85	17.46±18.87	0.14
		Non-colonial	38.80±9.69	41.85±12.49	44.49±8.64	0.58
	Biovolumes (µm3)	<99	30±19.85	33.54±11.40	53.19±21.29	0.21
		100–299	25.44±5.06	31.53±9.61	23.12±3.79	0.29
		300–599	15.31±9.51	12.89±5.45	6.79±5.26	0.16
		600–1499	17.85±7.54	13.26±5.70	11.17±9.83	0.43
		>1500	11.40±5.27	8.79±6.00	5.73±3.97	0.5
	Passy guilds	Low profile	42.46±21.27	37.71±11.55	65.79±25.38	0.23
		High profile	21.17±14.07	24.72±14.72	16.33±16.40	0.43
		Mobile	24.56±8.73	28.94±9.52	*11.96*±4.87 *	0.02
		Variable	3.98±5.34	3.63±1.75	1.82±1.41	0.43
**Diatoms - t30**	Pioneer forms	Non-pioneer	97.92±2.59	92.94±6.55	90.19±13.58	0.21
		Pioneer	2.08±2.59	7.06±6.55	9.81±13.58	0.21
	Growth forms	Adnate	3.36±2.00	3.77±1.97	2.81±1.54	0.59
		Pedunculate	15.95±10.15	20.15±9.08	18.75±8.16	0.79
		Colonial	46.09±21.86	44.63±16.44	35.63±21.39	0.66
		Non-colonial	34.60±14.60	31.46±8.68	42.81±14.93	0.43
	Biovolumes (µm^3^)	<99	26.98±15.17	23.96±4.47	22.92±10.21	0.84
		100–299	24.99±9.13	25.97±3.33	31.79±17.19	0.98
		300–599	18.69±5.35	15.95±3.08	14.79±3.26	0.33
		600–1499	17.01±6.22	20.04±4.84	17.31±7.11	0.49
		>1500	12.33±4.20	14.08±1.79	13.20±5.09	0.84
	Passy guilds	Low profile	35.08±14.64	34.80±7.39	31.97±12.85	0.69
		High profile	30.57±18.84	38.90±10.87	28.95±17.18	0.39
		Mobile	21.91±12.33	15.28±4.55	30.28±13.84	0.33
		Variable	3.64±3.29	3.13±2.52	2.05±1.48	0.87
**Diatoms - t15**	Pioneer forms	Non-pioneer	91.74±11.74	92.09±7.82	80.8±24.64	0.98
		Pioneer	8.26±11.74	7.91±7.82	19.20±24.64	0.98
	Growth forms	Adnate	3.57±2.32	2.96±1.73	2.35±1.96	0.77
		Pedunculate	20.33±9.53	20.54±7.31	20.91±14.17	0.92
		Colonial	40.17±21.35	39.66±16.74	19.60±14.68	0.23
		Non-colonial	35.93±13.41	36.83±10.88	57.15±20.56	0.12
	Biovolumes (µm^3^)	<99	23.99±9.55	21.69±7.10	27.39±21.94	0.98
		100–299	25.35±9.28	27.09±5.14	13.20±6.89	0.05
		300–599	15.53±4.34	13.89±3.68	36.45±33.16	0.55
		600–1499	23.32±3.35	24.52±5.88	15.25±8.95	0.12
		>1500	11.81±6.43	12.82±2.55	7.71±5.22	0.24
	Passy guilds	Low profile	34.38±11.26	29.46±9.31	32.32±22.83	0.87
		High profile	30.98±13.85	34.59±11.76	18.46±13.25	0.19
		Mobile	19.93±7.78	21.17±7.98	42.67±28.49	0.23
		Variable	3.90±1.30	3.60±0.84	2.23±2.11	0.43

Italics indicate that the metric is significantly different from the reference station (Up), stars indicate significant differences between Down 1 and Down 2.

**Benthic macroinvertebrates.** A higher number of taxa showing more than one reproductive cycle per year, and/or an asexual way of reproduction was observed downstream, in addition to a greater number of taxa showing a high maximal size (Table 4). Taxa with tegumentary respiration, burrowers, interstitial or permanently attached were also favoured downstream. Taxa without any form of resistance decreased in favour of taxa able to produce cocoons. Active dispersal (aerial and aquatic) decreased while aquatic passive dispersion increased (station Down 1). Feeding behaviours were also modified downstream whereas absorbers and deposit feeders, eating detritus, fine sediments and microorganisms dominated. As a result shredders, filter-feeders and scrapers (only at station Down 1) eating plants (alive or dead) or living microinvertebrates decreased. Concerning ecological traits, polysaprobic and eutrophic taxa increased downstream while xenosaprobic, oligotrophic and mesotrophic taxa decreased.

**Table 4:** Macroinvertebrate-related traits: calculation (mean and standard deviation) and differences between sites.

Trait	Modality	Up	Down1	Down2	*P* value
Maximal size	≤0.25 cm	0.10±0.05	*0.46*±*0.06*	*0.35*±*0.17*	0.03
	>0.25–.5 cm	22.41±13.39	*5.59*±*1.78*	*8.34*±*3.63*	0.02
	>0.5–1 cm	25.86±3.14	*12.7*±*3.93*	21.34±13.96	0.07
	>1–2 cm	27.51±8.47	*13.42*±*1.47*	*16.58*±*5.20*	0.03
	>2–4 cm	13.74±4.98	9.29±0.26	9.95±1.26	0.24
	>4–8 cm	8.15±6.13	*45.77*±*5.58*	*33.98*±*17.07*	0.03
	>8 cm	2.24±1.69	*12.76*±*1.55*	*9.47*±*4.76*	0.03
Life span	≤1 year	61.84±9.98	18.95±9.6	37.52±29.15	0.07
	>1 year	38.16±9.98	81.05±9.6	62.48±29.15	0.07
Number of reproductive cycles per year	<1	1.31±0.80	0.40±0.21	1.44±0.99	0.17
	1	50.29±12.22	*31.60*±*2.30*	36.65±6.28	0.03
	>1	48.40±12.71	*68*±*2.42*	61.91±7.10	0.03
Aquatic stage	egg	27.05±7.23	28.24±2.91	26.00±4.43	0.87
	larva	39.06±1.53	36.77±1.39	40.66±5.25	0.16
	nymph	19.5±7.46	8.80±4.73	13.46±9.28	0.21
	adult	14.4±3.91	26.18±3.15	19.87±9.74	0.07
Reproduction	ovoviviparity	17.59±11.17	*1.84*±*0.93*	*3.03*±*1.92*	0.02
	isolated eggs, free	2.20±1.23	5.63±0.72	4.65±2.15	0.08
	isolated eggs, cemented	4.82±3.42	2.77±0.34	3.55±1.57	0.66
	clutches, cemented or fixed	51.35±19.01	56.97±3.75	52.65±7.01	0.66
	clutches, free	15.30±10.60	9.21±4.95	13.20±9.26	0.66
	clutches, in vegetation	0.45±0.36	0.00±0.01	0.21±0.21	0.06
	clutches, terrestrial	4.72±2.37	3.32±1.52	7.68±4.33	0.19
	asexual reproduction	3.56±2.73	*20.26*±*2.47*	*15.03 ± 7.55*	0.02
Dispersal	aquatic passive	46.23±5.32	*80.11*±*6.02*	67.06±18.51	0.04
	aquatic active	21.28±3.39	*9.99*±*1.10*	*13.24*±*4.03*	0.02
	aerial passive	12.96±4.70	5.93±3.12	10.03±7.12	0.19
	aerial active	19.53±6.53	*3.96*±*1.86*	9.67±7.61	0.03
Resistance forms	eggs, statoblasts	3.56±2.57	*0.01*±*0.02*	4.23±4.93	0.02
	cocoons	6.53±5.02	*36.92*±*4.50*	*27.34*±*13.82*	0.03
	housings against desiccation	0±0	0±0	0±0	0.11
	diapause or dormancy	6.14±2.01	2.30±1.14	4.79±3.02	0.09
	none	83.77±5.91	*60.78*±*3.36*	*63.64*±*7.10*	0.02
Respiration	tegument	49.45±12.09	*90.58*±*2.47*	*79.90*±*12.31*	0.02
	gill	42.28±9.52	*9.01*±*2.45*	*18.56*±*12.40*	0.01
	plastron	6.75±6.77	*0.10*±*0.14*	*0.50*±0.32 *	0.01
	spiracle	1.52±0.97	0.30±0.37	1.03±1.20	0.17
	hydrostatic vesicle	0±0	0±0	0±0	
Locomotion	flier	2.63±2.62	*0.04*±*0.07*	*0.20*±0.12 *	0.01
	surface swimmer	0.06±0.03	0.01±0.01	0.06±0.09	0.16
	full water swimmer	13.21±6.51	8.57±1.55	11.24±5.76	0.55
	crawler	41.60±8.07	*7.52*±*3.65*	*17.59*±*13.13*	0.02
	burrower	9.11±4.94	*26.09*±*1.89*	*20.88*±*6.98*	0.02
	interstitial	19.50±6.97	*51.59*±*4.34*	*41.85*±*13.85*	0.02
	temporarily attached	13.85±11.44	6.00±1.07	8.03±3.02	0.24
	permanently attached	0.04±0.03	*0.17*±*0.02*	*0.16*±*0.03*	0.02
Food	microorganisms	3.59±2.26	*15.66*±*1.52*	*11.98*±*5.07*	0.02
	detritus (<1 mm)	26.85±4.00	*52.40*±*2.81*	*45.28*±*10.39*	0.02
	dead plant (≥1 mm)	9.97±1.38	*1.09*±*0.48*	*2.80*±*2.66*	0.02
	living microphytes	32.22±8.85	*23.28*±*0.40*	*24.64*±*2.43*	0.05
	living macrophytes	5.26±1.89	*1.42*±*0.68*	*2.64*±*2.12*	0.04
	dead animal (≥1 mm)	2.90±1.53	0.37±0.19	1.06±1.24	0.06
	living microinvertebrates	10.13±5.33	*2.08*±*1.11*	*3.52*±*2.48*	0.04
	living macroinvertebrates	9.06±2.61	3.70±1.63	8.05±5.04	0.07
	vertebrates	0.02±0.04	0±0	0.03±0.03 *	0.04
Feeding habitats	absorber	2.58±1.96	*14.78*±*1.80*	*10.95*±*5.53*	0.03
	deposit feeder	19.1±9.57	*64.99*±*5.44*	*52.16*±*16.83*	0.02
	shredder	21.91±6.67	*2.23*±*1.04*	*3.63*±*2.12*	0.02
	scraper	27.79±11.74	*8.61*±*1.66*	14.82±9.42	0.04
	filter-feeder	13.66±15.90	2.22±1.22	5.39±3.98	0.11
	piercer	1.29±0.89	1.35±0.71	4.47±2.95	0.11
	predator	11.67±4.08	4.62±2.30	6.90±4.90	0.13
	parasite	1.99±1.40	1.20±0.65	1.68±1.19	0.77
Transverse distribution	river channel	25.78±13.91	*11.33*±*1.10*	13.81±1.89	0.03
	banks, connected side-arms	35.64±2.91	*31.87*±*0.16*	*32.54*±0.65 *	0.01
	ponds, pools, disconnected side-arms	8.46±4.10	11.17±0.44	10.85±0.49	0.74
	marshes, peat bogs	3.89±2.17	6.02±0.29	5.59±0.27	0.13
	temporary waters	7.87±3.67	6.15±1.23	7.53±2.87	0.79
	lakes	16.29±5.28	*22.83*±*0.32*	*21.76*±*1.01*	0.02
	groundwaters	2.07±1.48	*10.64*±*1.30*	*7.92*±*4.00*	0.03
Longitudinal distribution	crenon	10.64±1.26	*12.32*±*0.23*	*12.10*±*0.37*	0.02
	epirithron	16.62±2.67	*13.79*±*0.12*	14.44±0.50	0.02
	metarithron	17.34±3.19	14.75±0.22	15.19±0.26	0.23
	hyporithron	17.60±3.63	16.13±0.39	16.21±0.51	0.92
	epipotamon	13.95±1.34	*16.91*±*0.40*	15.86±1.27	0.03
	metapotamon	9.71±2.42	*15.25*±*0.52*	13.76±1.85	0.03
	estuary	3.84±2.21	*1.15*±*0.49*	*1.61*±*0.96*	0.04
	outside river system	10.31±4.15	9.71±1.35	10.81±2.42	0.87
Altitude	lowlands	54.64±5.20	*67.68*±*3.27*	61.11±9.16	0.04
	piedmont level	25.87±4.61	*15.00*±*0.89*	*18.08*±*3.74*	0.02
	alpine level	10.23±2.29	8.48±1.27	11.10±3.52	0.31
Substrate	flags/boulders/cobbles/pebbles	16.27±3.38	11.96±0.41	13.61±2.00	0.06
	gravel	12.88±2.44	17.38±1.29	15.14±3.33	0.14
	sand	11.03±2.04	12.81±0.24	11.96±1.20	0.39
	silt	6.00±1.83	8.65±0.29	7.58±1.49	0.07
	macrophytes	16.22±1.08	12.87±0.54	14.91±2.72	0.09
	microphytes	2.62±1.09	3.10±0.14	2.61±0.61	0.43
	twigs/roots	8.53±3.66	*4.16*±*0.26*	5.32±1.80	0.03
	organic detritus/litter	5.82±2.00	5.98±0.08	5.95±0.15	0.98
	mud	5.92±3.35	*14.52*±*0.43*	*12.60*±*2.33*	0.02
Current velocity	null	16.62±8.61	24.75±0.67	22.73±1.55	0.05
	slow (<25 cm/s)	31.16±3.62	32.91±0.55	32.47±1.72	0.58
	medium (25–50 cm</s)	33.93±7.92	*26.26*±*0.38*	27.57±1.20	0.01
	fast (>50 cm/s)	18.30±1.78	16.08±0.26	17.22±2.05	0.15
Trophic status	oligotrophic	37.28±6.57	*31.68*±*0.75*	*32.73*±*0.76*	0.02
	mesotrophic	42.33±1.69	*38.78*±*0.25*	*38.66*±*0.27*	0.02
	eutrophic	20.39±5.89	*29.54*±*0.55*	*28.61*±*0.77*	0.01
Salinity (preferences)	freshwater	85.41±7.13	89.05±1.75	87.01±4.7	0.79
	brackish water	14.59±7.13	10.95±1.75	12.99±4.7	0.79
Temperature	cold (<15°C)	21.34±4.37	25.92±0.48	23.81±2.46	0.29
	warm (>15°C)	10.53±2.91	*16.23*±*0.17*	14.71±2.10	0.03
	eurythermic	68.12±6.30	*57.85*±*0.51*	61.48±4.55	0.03
Saprobity	xenosaprobic	9.25±2.05	*3.25*±*1.08*	*5.42*±*3.36*	0.04
	oligosaprobic	27.68±1.76	23.67±0.83	25.40±2.44	0.07
	β-mesosaprobic	40.95±5.03	40.74±0.98	40.51±1.69	0.84
	α-mesosaprobic	18.47±4.05	*23.78*±*0.73*	21.45±2.62	0.03
	polysaprobic	3.64±2.14	*8.56*±*0.25*	*7.22*±*1.51*	0.01

Italics indicate that the metric is significantly different from the reference station (Up), stars indicate significant differences between Down 1 and Down 2.

**Macrophytes.** The different floristic group proportions were clearly modified downstream (Table 5). Station Down 1 presented more than 99% of heterotrophic forms, hence phanerogram-related traits were not reliable for its description. At station Down 2 the phanerogams did not recover the upstream reference status, allowing the installation of filamentous algae. At this site, taxa were preferentially annual, with asexual reproduction mainly by fragmentation and sexual reproduction based on higher numbers of seeds, with entomophilous dispersion. These taxa showed larger emergent leaves, related to a higher morphological index (large size).

**Table 5:** Macrophyte-related traits: calculation (mean and standard deviation) and differences between sites.

Trait	Modality	Up	Down1	Down2
Floristic group	Algae	3.63±2.84	*0.02*±*0.03*	*50.84*±33.98 *
	Heterotroph	0±0	*99.58*±*0.53*	0±0
	Bryophyte	35.00±5.67	*0.36*±*0.51*	35.55±27.43
	Phanerogam	61.38±2.83	*0.03*±*0.05*	*13.61*±6.54 *
Growth form	Anchored, floating leaves	33.23±0.06	NA	31.31±2.41
	Anchored, submerged leaves	33.47±0.01	NA	34.34±1.21
	Anchored, emergent leaves	4.25±3.27	NA	*9.02*±*5.42*
	Anchored, heterophylly	29.05±3.31	NA	*25.32*±*4.21*
Vertical shoot architecture	Single apical growth point	2.92±2.19	NA	0±0
	Single basal growth point	12.26±10.39	NA	14.98±1.26
	Multiple apical growth point	84.82±8.21	NA	85.02±1.26
Leaf type	Capillary	0.52±0.10	NA	0±0
	Entire	99.48±0.10	NA	100±0
Leaf area	Small (<1 cm^2^)	78.01±9.83	NA	*64.86*±*19.20*
	Medium (1–20 cm^2^)	9.02±2.27	NA	11.93±6.70
	Large (20–100 cm^2^)	12.76±7.86	NA	*20.01*±*7.96*
	Extra-large (>100 cm^2^)	0.21±0.30	NA	*3.21*±*4.53*
Morphology index (score)	3–5	44.91±1.89	NA	*34.28*±*6.89*
	6–7	46.70±3.12	NA	49.94±13.26
	8–9	7.93±5.19	NA	*15.53*±*6.72*
	10	0.45±0.18	NA	0.25±0.35
Mode of reproduction	Rhizome	7.40±3.34	NA	9.39±3.58
	Fragmentation	40.25±5.89	NA	*36.49*±*4.78*
	Stolons	5.67±4.53	NA	*10.05*±*4.51*
	Seeds	46.67±1.98	NA	44.06±3.31
Number of reproductive organs per year per individual	Medium (10–100)	60.85±4.79	NA	*54.36*±*7.42*
	High (100–1000)	39.15±4.79	NA	*45.64*±*7.42*
Perrenation	Annual	29.49±1.87	NA	*34.74*±*15.65*
	Biennial / Short lived perrenial	0.08±0.11	NA	0.89±1.26
	Perennial	70.43±1.98	NA	*64.38*±*14.4*
Gamete vector	Wind	43.93±2.65	NA	43.32±0.28
	Water	18.39±0.60	NA	*16.69*±*2.58*
	Insect	0.14±0.20	NA	*4.03*±*4.82*
	Self	37.54±1.85	NA	35.96±1.96
Body flexbility	Low (<45°)	0.08±0.11	NA	0±0
	Medium (45–300°)	32.81±0.12	NA	*37.33*±*4.99*
	High (>300°)	67.11±0.01	NA	*62.67*±*4.99*
Leaf texture	Soft	37.49±0.91	NA	37.09±0.81
	Rigid	3.47±0.92	NA	4.36±2.04
	Waxy	21.54±0.90	NA	21.46±0.42
	Non-waxy	37.49±0.91	NA	37.09±0.81
Period of production of reproductive organ	Early (March-May)	31.34±0.96	NA	*28.45*±*3.97*
	Mid (June-August)	36.07±2.94	NA	38.68±2.41
	Late (August-September)	31.57±1.28	NA	32.87±1.56
	Very late (post- September)	1.02±0.70	NA	0±0
Fruit size	<1 mm	0±0	NA	0±0
	1–3 mm	87.74±10.39	NA	85.02±1.26
	>3 mm	12.26±10.39	NA	14.98±1.26

Italics indicate that the metric is significantly different from the reference station (Up), stars indicate significant differences between Down 1 and Down 2. NA: Metric calculation not possible.

**Fish.** Functional metrics were not applicable in station Down 1, where only one species (2 individuals) was found. At station Down 2 the biomass and abundance of invertivorous species decreased in favour of omnivores (Table 6). Pelagic species became dominant over benthic ones.

**Table 6:** Fish-related traits: calculation (mean and standard deviation) and differences between sites.

	Trait	Up	Down1	Down2
**Biomass**	Invertivorous	55.65	NA	*25.00*
	Omnivorous	44.35	NA	*75.00*
	Other	0.00	NA	0.00
	Phytophilic	0.00	NA	0.00
	Lithophilic	44.35	NA	*100.00*
	Mixed	55.65	NA	*0.00*
	Benthic	74.78	NA	*16.70*
	Pelagic	25.22	NA	*83.33*
**Abundance**	Invertivorous	21.29	NA	*14.52*
	Omnivorous	78.71	NA	*85.48*
	Other	0.00	NA	0.00
	Phytophilic	0.00	NA	0.00
	Lithophilic	78.71	NA	*90.32*
	Mixed	21.29	NA	*9.68*
	Benthic	43.78	NA	*8.06*
	Pelagic	56.22	NA	*91.94*

Italics indicate that the metric is significantly different from the reference station (Up). NA: Metric calculation not possible.

## Discussion

### The importance of biology for the assessment of ecological status

PCA performed on environmental data discriminated upstream from downstream conditions, but sites Down 1 and Down 2 were not well distinguished, in contrast to results obtained with biological data (especially for macro-invertebrates). A high seasonal variation can also be noted, with different dilution conditions according to whether the flow was low or high.

Moreover, several toxicants known to be released in the plant effluents were not detected by physicochemical analysis. Thus, such analysis does not seem to be particularly reliable in the case of the River Luzou: to characterize intermittent pollution, measurements should be performed exactly during the releases, or by high resolution analyses. The problem of toxicity related to cocktail effects and degradation compounds can also hardly be tackled in this way. In this context, biological related metrics are potentially more informative than chemical analyses. Seasonal variations in community structure (Fig. 3) were also observed, and the different metrics used allowed the nature, and intensity, of the pollution present in the River Luzou to be highlighted.

### Temporal scales of biological responses

The seasonal variations observed in the PCA performed on environmental data were correlated to changes in diatom responses over time. Although quantitative measurements indicated decreasing diatom biomass from up- to downstream whatever the sampling season, the number of cells settled was up to 15 fold higher in warmer conditions compared to winter (data not shown). The seasonality in water contamination was highlighted by, e.g., higher percentages of teratologies (up to 3% on natural substrates) and of the species *Achnanthidium minutissimum* downstream in Autumn, indicating toxic pollution [45]–[46].

Diatoms have fast growth rates (from hours to days), and thus respond very quickly to variations in their environment. BQE with longer life span reflect more averaged water quality on different time scales. Seasonal patterns were thus less pronounced for macrophytes and macroinvertebrates that integrated global quality over the year. Ultimately, fish responses were expected to reveal environmental conditions on the longer term (years).

### Taxonomic metrics reveal pollution intensity

Richness, diversity and equitability indices are classically used to evaluate ecological status of water and their decline is indicative of a disturbed environment [47]. Except for diatoms, these metrics clearly decreased for all the biological compartments studied, at station Down 1, which is in accordance with the literature [16], [48]–[49]. They recovered to variable extents at Down 2, indicating weaker biological impact likely due to lower toxicant availability (by dilution and/or toxicant degradation). For diatoms, the phenomenon observed contrasted with the literature, rather reporting a decrease of these taxonomic metrics in toxic conditions [45], [50]. For this BQE in our study, richness, diversity and equitability indices were not relevant to highlight the toxic impact of pollution (see below).

### Biological indices rather indicate the nature of pollution

Biological indices except IBD and IPS drastically decreased downstream, due to the presence of numerous tolerant taxa for IBMR and IBGN (*Sphaerotilus* sp., *Oligochaeta* and *Chironomidae*), or to the lack of fish populations for IPR. SPEARorganic index being negatively correlated to toxicants (icides, surfactants, petrochemicals) [11], its decrease downstream was also consistent. In contrast IPS and IBD scores remained good due to the high proportion of *Achnanthidium minutissimum* (up to 60%). However, the occurrence of abnormal forms, mainly affecting *A. minutissimum*, clearly characterized downstream conditions. Above 1%, this rate of occurrence is considered to reflect the impact of toxic pollutants on diatom communities [45]–[46]. Moreover, recent works also suggested that, due to its pioneering character, *A. minutissimum* was indicative of toxic pollution [45]. Therefore, the use of diatom-based biological indices to highlight toxic pollution is not recommended, but a careful analysis of community composition (as used for index calculations) can also provide information regarding the nature of the contamination.

Additionally, quantitative metrics (decrease of chlorophyll *a* concentrations and diatom density downstream) were consistent with Morin et al. [45], and with Wang et al. [51] who demonstrated that a derivative of aniline could inhibit adhesion in certain diatom species. The percentage of live cells tended to decrease downstream, as already reported by Stevenson and Bahls [52] and Gillet et al. [53] who examined whether the percentage of live diatoms in periphyton communities could be used as a metric of human disturbance in streams and rivers.

Periphytic biomass (dry weight and ash-free dry mass) tended to increase downstream, whereas diatom cell numbers and chlorophyll a did not. This unexpected result can perhaps be related to the periphyton becoming more heterotrophic, which would be consistent with the massive development of *Sphaerotilus* sp. observed at Down 1 in the macrophytic community. One could also hypothesize that rivers from Landes ecoregion, characterized by naturally acidic waters and nutrient depletion, represent a particular ecosystem where artificial eutrophication (concomitant to industrial release) can enhance the periphyton growth and richness. Other recent works [54] reported reduced impacts on biomass of metal toxicity in acid-adapted biofilms. These phenomena may explain the typical quantitative response of phytobenthos towards toxic pollution.

### Survival strategies under high pollution

Functional metrics illustrated how the ecosystem of the River Luzou changed under toxic pollution. According to the results, many modifications concerning the four BQE were convergent, like reproduction and colonization strategies, or the trophic regime. Southwood [55] wrote that those physiological adaptations were typically found in impaired sites, as they induce tolerance to harsh conditions.

First, when escaping pollution, as fish attempt to do, was not possible, resistant taxa became dominant (*Sphaerotilus* sp., *Oligochaeta* and *Chironomidae*), with production of cocoons as extreme resistance forms for macroinvertebrates.

Early colonizers with ruderal strategies [56] were also favoured, like the diatom *Achnanthidium minutissimum* which reached an abundance of up to 60% downstream. Morin et al. [57] already observed this high abundance of *A. minutissimum* under toxic conditions, implying a thinning of the biofilm by loss of high-profile taxa (according to Passy [22], high profile guild reaches a maximum in nutrient-rich sites and in conditions of low flow disturbance). *Achnanthidium minutissimum* was probably less disfavoured thanks to its small size reducing the exposure time to toxicants [58], or its adnate posture. Macrophyte communities evolved towards the predominance of annual taxa producing a great number of reproductive organs per year and per individual (e.g. *Berula erecta*, *Scirpus fluitans* or *Sparganium erectum*), ensuring a rapid spread across the river. Macroinvertebrates' high colonization ability was provided by the combination of a greater number of reproductive cycles and dispersion by drift, those two strategies being already reported in the literature [59]. Drift is an important way of species dissemination and recolonisation of river systems by lotic macroinvertebrates [60], and is known to increase with chemical disturbance [61].

Finally, taxon sizes showed opposite trends. Diatom taxa with lower biovolumes were favoured, whereas bigger macroinvertebrate taxa, or macrophytes with large emergent leaves, increased downstream. This, however, resulted from the same strategy of organisms facing toxicants. Indeed, a decrease of diatom cell sizes are generally observed in environments exposed to toxic pollutions: reduction of size expresses increased vegetative multiplication, and reduced sexual reproduction (not measured here, but concordant with traits of other compartments: higher reproduction rates and asexual reproduction), and selection of smaller species (reducing uptake of toxicants into the cell).

### Toxic pollution also drives indirect changes across the trophic web

Our results highlighted changes in feeding habits, which represent an important aspect of the community trophic structure modifications. Summarizing, the reference ecosystem (Up) showed balanced communities composed of species typical of the Landes ecoregion [62]. The different compartments are driven by complex interactions. Primary producers provide food resources to primary consumers (like macroinvertebrates and fish feeding on phytobenthos) but also refuge and habitat and/or egg-laying substrates (especially macrophytes). The modifications observed in these communities (disappearance or sharp decline of phanerogams, biofilm thinning) represent a real impact on invertebrates and may explain the changes observed downstream. In particular, in such streams like the river Luzou where there is a poor diversification of abiotic substrates, macrophytes not only provide a food source, but also a shelter for invertebrates [63]. Macroinvertebrate structure and biomass are also modified by the presence or absence of predators. With high pollution (Down 1), biomass of primary producers decreased to be replaced by filamentous heterotrophs, affecting the subsequent components in a cascade. Aside from some potential direct toxicity (according to the toxicants mode of action), macroinvertebrates were probably driven by the resource, selecting absorbers and deposit feeders feeding on detritus and microorganisms in accordance with Archaimbault et al. [60] and Schultheis et al. [64]. Moreover, the decline of macroinvertebrate communities may have forced invertivorous macroinvertebrates and fish to leave the site. Further downstream (Down 2), primary producers tended to diversify, as well as primary consumers and predators. This partial recovery thus reflected both direct (reduction of toxic pressure, slight increase in nutrient availability) and indirect (return to a more balanced ecosystem) improvement of the ecosystem.

## Conclusions

In conclusion, our study revealed that in a context of multiple contaminants, of pulse inputs, of complex cocktails, and/or of release of unknown substances, a combination of different biological measurements, from different aquatic communities, can be much more informative than punctual physicochemical analysis and single biotic indices.

Under such strong and diverse anthropogenic pressure, a multicompartment approach allows the integrated observation of community trajectories towards adaptation, accounting for the complex biotic relationships in aquatic ecosystems. Colonization strategies, reproduction and trophic regimes seem to be key indicators of this adaptation.

A further step would be to continue this multi-compartment survey during restoration programmes, to identify the behaviour, and/or biological elements that tend to recover more rapidly. Data from the literature suggest that mobile organisms would have the greatest ability to recolonize sites after water quality improvement. The time necessary to reach complete recovery of the ecosystem when pollution ceases is also likely to be very variable among biological compartments and is an important component to be determined in the context of the implementation of the Water Framework Directive.

## Supporting Information

Checklist S1 **ARRIVE guidelines.**
(DOC)
